# Supplementary material for: Promoting positive parenting and mental wellbeing in Hong Kong Chinese parents: A pilot cluster randomised controlled trial
Source: PLoS One. 2022 Jul 20;17(7):e0270064. doi: 10.1371/journal.pone.0270064 (PMC9299310; doi:10.1371/journal.pone.0270064)
Supplement: S1 Protocol — (PDF) [file pone.0270064.s001.Pdf]

STUDY PROTOCOL of Joyful Parenting Pilot Project RCT Study

1. General Information

- Protocol title: **Community-based Mental Wellness Project for Adolescents and Adults: Joyful Parenting Pilot Project RCT Study**
- Name of the sponsor: Health Care and Promotion Fund, Food and Health Bureau
- Name of the principal investigator: Dr. Sun Yuying, Grace
- Title of the principal investigator: Post-doctoral Fellow, School of Public Health, Li Ka Shing Faculty of Medicine, The University of Hong Kong
- Name of the Co-investigators: Dr. Wang Man Ping, Kelvin; Dr. Ho Sai Yin, Daniel; Prof. Lam Tai Hing
- NCT number: NCT03282071
- Document date: Version no.02 dated 2017.06.01

2. Background

This project adopts the five elements of Seligman's well-being theory (Seligman, 2002 & 2011). PERMA includes five pillars of well-being that can help people reach a life of fulfillment, happiness and meaning: Positive emotions (P), Engagement (E), Positive relationships (R), Meaning (M) and Accomplishment (A). "Joyful@HK" was launched as a territory-wide intervention and mental health promotion campaign in Hong Kong. Three main elements are being advocated: sharing, mind, and enjoyment (SME). The elements being promoted in Joyful@HK reflect the principles of positive psychology and the positive interventions supported by evidence of efficacy (Sheldon and Lyubomirsky, 2006; Sin and Lyubomirsky, 2009; Gander et al., 2012).

The Joyful Parenting Pilot Project is organized by the Hong Kong Family Welfare Society and is funded by Health Care and Promotion Fund. The main targets are 120 parents and at least 240 family members. The project will organize two-session interactive talks for parents to promote praise and appreciation, positive thinking, enjoyable parenting (Mind) and knowledge of adult mental health problem (in particular the mixed anxiety and depressive disorder) and mental well-being; and one family gathering activity to promote and practice quality family communication (Enjoyment and Sharing). One wisdom sharing session will be organized for social workers and service practitioners to promote best practice at the end. School of Public Health, The University of Hong Kong will evaluate the effectiveness of the Joyful Parenting Pilot Project.

Evidence of effectiveness from our previous project: The Effective Parenting Programme conducted from August 2010 to June 2012 with collaboration of Caritas- Hong Kong that aimed to increase parent's use of emotion management techniques, improve their emotional well-being, parent-child relationship and family 3Hs. Low brief interventions were delivered by the social workers and randomized controlled trial was used to evaluate the programme

effectiveness. A total of 412 parents with children in Primary 1-3 participated in the programme. At 3, 6 and 12-month follow up, the intervention group reported greater increases in the use of emotion management strategies during parent-child interactions (Cohen's f = 13.5- 32.67, all p< 0.001), with small to medium effect size (0.19- 0.29), and lower negative affect (Cohen's f = 6.83- 14.87) and greater positive affect (Cohen's f = 5.20- 14.15), subjective happiness (Cohen's f = 15.62- 23.68), satisfaction with the parent-child relationship (Cohen's f = 6.55-15.22), and family harmony (Cohen's f = 17.25-18.43), compared to the control group, with small to medium effect size (0.12- 0.26). (Fabrizio, C. S. et al., 2015) It suggests such programmes can provide a successful, sustainable, preventive, behaviorally-based emotion management model for Chinese mothers.

3. Objectives

3.1 Process evaluation:

- To assess the satisfaction level of parents towards the Joyful Parenting Pilot Project
- To collect community partners' feedbacks and components on improving future community- based mental wellness programmes

3.2 Outcome evaluation:

- To promote the concept of Sharing, Mind and Enjoyment (SME); to increase the knowledge and understanding about mental health for adolescents and adults.

4. Selection and Withdrawal of subjects

4.1 Subject Inclusion criteria

(1) Parents:

- Parents aged 18-59 (including single parents)
- Chinese speaking
- Able to complete questionnaires/ focus groups

(2) Their family members:

- Family members of parents who participated in the Joyful Parenting Pilot Project
- Chinese speaking
- Able to complete questionnaires/ focus groups

(3) Community partners

- Staffs of Family Service Units under Hong Kong Family Welfare Society in partnership with HKU research team to organize, plan, or implement the project
- Adults aged 18 or above
- Chinese speaking
- Able to complete questionnaires/ individual in-depth interviews

4.2 Subject exclusion criteria

- Those who cannot read Chinese
- Those who suffered from severe mental illness

5. Research design

5.1 Subject recruitment

Four family service units under Hong Kong Family Welfare Society will be randomized into two intervention family service units and two control family service units. Cluster randomized controlled trial design will be adopted.

Four family service units of Hong Kong Family Welfare Society will recruit parents and their family members who are eligible to the study under inclusion criteria as intervention subjects and control subjects. Two intervention family service units will be responsible for the implementation of the intervention and recruitment of intervention subjects, while two control family service units will be responsible for the recruitment of control subjects of the study.

Staffs of Hong Kong Family Welfare Society as our community partners will also be recruited for the individual in-depth interviews in order to evaluate the community engagement approach of this project to improve future public health practice.

5.2 Intervention arms

The evaluation will include 2 arms (each having 60 parents and at least 120 family members)

- **2 Intervention family service units:** Two-session interactive talks and one family gathering activity
- **2 Waitlist control family service units:** Waitlist control receiving no intervention during evaluation period. Preferably these family service units should have similar characteristics with the intervention family service units. They will be provided with the intervention after the evaluation period.

5.3 Assessments

- T1: Before the intervention (baseline)
- T2: Immediate post intervention
- T3: One-month follow-up after baseline
- T4: Three-month follow-up after baseline
- The above timing is for the intervention family service units. Each control family service units will complete the T1, T3 and T4 assessments about the same time as the matched intervention family service units.

5.4 Process Evaluation (Table 1)

The opinion of parents and their family members in the intervention group will be assessed quantitatively (questionnaire surveys) and qualitatively (focus group interviews)

a) T2 (Immediate post intervention)

The satisfaction, ratings and opinions on the interactive talks of parents in the intervention group will be assessed quantitatively (questionnaire surveys)

- Parents
  - Quantitative: questionnaire survey in family service units
    - All parents from the intervention family service units: n=60

b) T3 (1-month follow-up after baseline)

The satisfaction, ratings and opinions on the interactive talks of parents in the intervention group will be assessed quantitatively (questionnaire surveys). Process evaluation among parents at T3 is embedded into outcome evaluation (refer to outcome evaluation below)

- Parents
  - Quantitative: questionnaire survey in family service units
    - All parents from the intervention family service units: n=60

c) T4 (3-month follow-up after baseline)

The satisfaction, ratings and opinions on family gathering activities will be assessed quantitatively (questionnaire surveys) and qualitatively (focus group interviews). Quantitative evaluation among parents and their family members at T4 is embedded into outcome evaluation (refer to outcome evaluation below)

- Parents
  - Quantitative: questionnaire survey in family service units
    - All parents from the intervention family service units: n=60
  - Qualitative: focus group interviews in family service units
    - At least 4 groups will be conducted for the parents and/or their family members
    - Each group has 8- 12 participants. (n=32-48)
    - The interviews will be audio-recorded
    - Each interview is conducted by 1 interviewer and 1 assistant

- Family members
  - Quantitative: questionnaire survey in family service units
    - All family members from the intervention family service units: n=120

- Community partners
  - Qualitative: in-depth individual interviews in family service units

- At least 4 individual interviews will be conducted for the community partners (n=4)
- The interviews will be audio-recorded
- Each interview is conducted by 1 interviewer and 1 assistant

Table 1. Outline of process evaluation

Target	Timeline	Process evaluation (intervention group)	
		Quantitative	Qualitative
Parents	T2	- Immediately after 1st session interactive talk<br>- 2 family service units<br>- 60 parents (30 parents from each service unit)	N/A
	T3	- Immediately after 2nd interactive talk<br>- 2 family service units<br>- 60 parents (30 parents from each service unit)	N/A
	T4	- Immediately after family gathering activity<br>- 2 family service units<br>- 60 parents (30 parents from each service unit)	- At least 4 focus groups<br>- 8-12 participants per group<br>- n=32-48
Family members	T4	- Immediately after family gathering activity<br>- 2 Family service units<br>- 120 family members	N/A
Community partners	T4	N/A	- At least 4 individual interviews<br>- n=4

5.5 Outcome Evaluation (Table 2)

Background, sharing, mind and enjoyment related behavioral indicators, family relationship, well-being and subjective changes of parents in intervention group and control group will be assessed quantitatively (questionnaire survey)

a) T1 (baseline) evaluation:
   - Parents

- Quantitative: questionnaire survey in family service units
  - All parents from the intervention family service units: n=60
- Items: background, mental health knowledge, SME-related behavioral indicators, family relationship, mental well-being

b) T2 (immediate post intervention):
- Parents
  - Quantitative: questionnaire survey in family service units
    - All parents from the intervention family service units: n=60
  - Items: background, awareness and attitude change, intention of behavioral change

c) T3 (1-month after baseline) evaluation
- Parents
  - Quantitative: questionnaire survey in family service units
    - All parents from the intervention family service units: n=60
  - Items: background, mental health knowledge, SME-related behavioral indicators, family relationship, mental well-being, subjective changes

d) T4 (3-month after baseline) evaluation (embedded in the above T4 process evaluation)
- Parents
  - Quantitative: questionnaire survey in family service units
    - All parents from the intervention family service units: n=60
  - Items: background, mental health knowledge, SME-related behavioral indicators, family relationship, mental well-being, subjective changes

e) Qualitative outcome evaluation
- Parents
  - Qualitative focus group interviews in family service units
    - At least 4 groups will be conducted for the parents
    - Each group has 8- 12 participants. (n=32-48 participants)
    - The interviews will be audio-recorded
    - Each interview is conducted by 1 interviewer and 1 assistant
- Community partners
  - Qualitative in-depth individual interviews in family service units
    - At least 4 individual interviews will be conducted for the community partners
    - The interviews will be audio-recorded
    - Each interview is conducted by 1 interviewer and 1 assistant

Table 2. Outline of outcome evaluation

Target	**Outcome evaluation**	
	**Intervention Group**	**Control Group**
Parents	- T1, T2, T3, T4 surveys<br>- 2 Family Service Units<br>- 60 parents<br>(30 parents from each service unit)<br>- T2, T3, T4 survey includes process evaluation	- T1, T3, T4 surveys<br>- 2 Family Service Units<br>- 60 parents<br>(30 parents from each service unit)
Family members	- 2 Family Service Units<br>-At least 120 family members<br>- T3 survey includes process evaluation<br>- 4 Focus groups (n=32-48)<br>- T4 survey includes process evaluation	- 2 Family Service Units<br>-At least 120 family members
Community partners	Individual in-depth interviews<br>- 2 Family service units	Individual in-depth interviews<br>- 2 Family service units

6. **Statistics**

Descriptive findings will be presented as means and proportions with 95% confidence interval. Standard statistical methods for RCT (e.g. intention to treat) and subgroup analysis will be set a priori in the protocol which will be registered in clinicaltrial.gov. Missing data will be handled using ITT, LOCF or multiple imputation depending on the patterns of missing data. Clustering effect if any will be handled with statistical models (e.g. multi-level analysis) and regression models will be used to estimate the effect sizes of interventions.

7. **Ethics**

An invitation letter detailing the study will be given to the participants, explaining the arrangements of the voluntary surveys. Participants may discontinue participation at any time

without penalty or loss. The voluntary nature of participation will be emphasized and passive consent for participation will be sought.

8. Data handling and data keeping

All data collected during the course of research will be kept strictly confidential. Data will be handled and stored in locked cabinet and password-protected computers during and after completion of the study. The investigator (Dr. Sun Yuying) and her appointed staff will be responsible for safekeeping the data. Only the research team and appointed staff will have access to the data during/after the study. The personal data will be kept for 3 years from the end of the study.

9. Compliance with the ICH-GCP

This protocol has complied with the ICH-GCP.

10. References

Fabrizio, C. S., Lam, T. H., Hirschmann, M. R., Pang, I., Yu, N. X., Wang, X., & Stewart, S. M. (2015). Parental emotional management benefits family relationships: A randomized controlled trial in Hong Kong, China. Behaviour Research and Therapy, 71, 115-124.

Gander, F., Proyer, R.T., Ruch, W. et al., 2012. Strength-based positive interventions: Further evidence for their potential in enhancing well-being and alleviating depression. Journal of Happiness Studies, 14(4), pp.1241-1259.

Seligman M. E. P. (2002). Authentic Happiness. New York, NY: Simon and Schuster.

Seligman M. E. P. (2011). Flourish. New York, NY: Free Press.

Sheldon, K.M. and Lyubomirsky, S., 2006. How to increase and sustain positive emotion: The effects of expressing gratitude and visualizing best possible selves. The Journal of Positive Psychology 1(2), pp.73-82.

Sin, N.L. and Lyubomirsky, S., 2009. Enhancing well-being and alleviating depressive symptoms with positive psychology interventions: a practice-friendly meta-analysis. Journal of Clinical Psychology, 65(5), pp.467-487.
