# Supplementary material for: Promoting positive parenting and mental wellbeing in Hong Kong Chinese parents: A pilot cluster randomised controlled trial
Source: PLoS One. 2022 Jul 20;17(7):e0270064. doi: 10.1371/journal.pone.0270064 (PMC9299310; doi:10.1371/journal.pone.0270064)
Supplement: S1 File — (DOCX) [file pone.0270064.s003.docx]

**S3** **Programme Rundown**

| Time | Content |
| --- | --- |
| Baseline questionnaire | **Baseline outcome evaluation (before the 1^st^ interactive talk)** |
| **1^st^ interactive talk** |  |
| 20mins | 1. The symptoms of Mixed Anxiety and Depressive Disorder 2. How to seek help for emotional disturbances 3. The importance of positive mind and positive discipline 4. Use positive discipline method to promote positive parent-child relationship |
| 5mins | Introduction of the SME concept   - Sharing: appreciate, praise and encourage children - Mind: explore children’s strengths, understand children in the positive way - Enjoyment: enjoy the interaction with children |
| 10mins | 1. Group discussion: Parents discuss about their experiences of giving praise to children 2. Instructors: praise could bring positivity and build a positive environment |
| 15mins | Experience parenting/family games  Game 1: Slapjack (a simple standard-deck card game)   - First round: remind participants to enjoy the game - Second round: appreciate the team members’ performance - Third round: parents try to praise each other and notice their feelings |
| 10mins | Short talk: how to practice appreciation, praise and encouragement   - give detailed and specific praise by using more adjective words (specific praise) - praise for their efforts made in the process even if they fail (process praise) - praise for improvements made rather than focusing on the achievements they made (give outcome praise in the right way) |
| 10mins | Experience parenting/family games  Game 2: Paper basketball  Using the skills to give praise or show appreciation (focus on specific praise) |
| 10mins | Experience parenting/family games  Game 3: Which household appliance  Using the skills to give praise or show appreciation (focus on process praise) |
| 10mins | Parents write down eight strengths of their children and share with the other parents in the group |
| 10mins | Summary |
| 10mins | - Reminders to complete worksheet - Complete process evaluation |
| After 1 month | **1-month outcome evaluation** (first follow-up before the 2^nd^ interactive talk) |
| **2^nd^ interactive talk** |  |
| 10mins | Warm-up game: transfer hula hoop  Remind the participants to complete the task cooperatively, encourage and praise the other members in the process |
| 35mins | 1. Homework review 2. Group discussion: discuss about their experiences in the past month  - What type of parenting/family game they have arranged? - Did they give praise and encouragement to children? - How did the children feel when parents praise them? How did the parents feel?  1. Review the skills of praise |
| 25mins | Experience parenting/family games  Game 1: Mono block lego  Remind the participants to complete the task cooperatively, encourage and praise the other members in the process |
| 20mins | Experience parenting/family games  Game 2: Role play  To let the participants experience how to give appropriate praise and observe children’s responses to different praise |
| 15mins | Summary  Review again the SME: sharing, mind and enjoyment |
| 15mins | - Remind parents to do more practice at home - Complete process evaluation |
| After 3 months | **3-month outcome evaluation** (second follow-up before the family gathering activity) |
| **Family gathering activity (2 hours)** | Family games  Lunch gathering |
