# Supplementary material for: Promoting positive parenting and mental wellbeing in Hong Kong Chinese parents: A pilot cluster randomised controlled trial
Source: PLoS One. 2022 Jul 20;17(7):e0270064. doi: 10.1371/journal.pone.0270064 (PMC9299310; doi:10.1371/journal.pone.0270064)
Supplement: S1 Table — (DOCX) [file pone.0270064.s004.docx]

# S3. Supplementary Table

# The comparison between intervention group (n=75) and control group (n=69).

| Items | Phase | BMD (95%CI) ^a^ | Cohen’s d (95% CI) | P-value ^b^ |
| --- | --- | --- | --- | --- |
|  |  | Intervention vs Control | Intervention vs Control |  |
| Praise (days) | 1 month | 0.67 (-0.63, 1.97) | 0.14 (-0.13, 0.41) | 0.310 |
|  | 3 months | -0.08 (-1.53, 1.36) | -0.02 (-0.37, 0.33) | 0.911 |
| Praise (times) | 1 month | 3.75 (-1.45, 8.95) | 0.19 (-0.07, 0.45) | 0.158 |
|  | 3 months | 0.60 (-3.70, 4.90) | 0.03 (-0.21, 0.28) | 0.785 |
| Appreciation | 1 month | -0.16 (-1.29, 0.97) | 0.02 (-0.37, 0.28) | 0.777 |
|  | 3 months | 1.07 (0.18, 1.97) | 0.37 (0.06, 0.69) | 0.019 |
| Enjoyment | 1 month | 0.15 (-1.10, 1.40) | 0.05 (-0.35, 0.44) | 0.812 |
|  | 3 months | 0.86 (-0.12, 1.84) | 0.32 (0.003, 0.63) | 0.047 |
| Subjective happiness | 1 month | 0.39 (0.13, 0.64) | 0.36 (0.12, 0.59) | 0.003 |
|  | 3 months | 0.44 (0.19, 0.69) | 0.44 (0.19, 0.70) | <0.001 |
| Well-being | 1 month | 0.28 (-0.70, 1.26) | 0.07 (-0.18, 0.33) | 0.572 |
|  | 3 months | 1.27 (0.22, 2.31) | 0.30 (0.05, 0.55) | 0.018 |
| Personal health | 1 month | 0.40 (-0.08, 0.87) | 0.19 (-0.04, 0.41) | 0.100 |
|  | 3 months | 0.65 (0.12, 1.18) | 0.33 (0.0.6, 0.59) | 0.016 |
| Personal happiness | 1 month | 0.32 (-0.13, 0.76) | 0.16 (-0.06, 0.37) | 0.163 |
|  | 3 months | 0.70 (0.21, 1.20) | 0.36 (0.11, 0.62) | 0.005 |
| Family health | 1 month | 0.43 (-0.02, 0.88) | 0.23 (-0.01, 0.46) | 0.059 |
|  | 3 months | 0.65 (0.14, 1.15) | 0.36 (0.08, 0.64) | 0.012 |
| Family happiness | 1 month | 0.51 (0.03, 0.99) | 0.26 (0.02, 0.51) | 0.036 |
|  | 3 months | 0.78 (0.28, 1.28) | 0.43 (0.15, 0.70) | 0.002 |
| Family harmony | 1 month | 0.30 (-0.17, 0.78) | 0.15 (-0.08, 0.38) | 0.213 |
|  | 3 months | 0.72 (0.24, 1.20) | 0.37 (0.12, 0.62) | 0.003 |
| Family relationship | 1 month | 1.17 (-0.04, 2.38) | 0.24 (-0.01, 0.49) | 0.059 |
|  | 3 months | 2.25 (0.91, 3.59) | 0.45 (0.18, 0.52) | 0.001 |

BMD, between-group mean difference; CI, confidence interval.

^a^ The differences between two groups at 1 month or 3 months were adjusted for the baseline of the corresponding variables.

^b^ *p* values were calculated using multilevel mixed-effects linear regression model.
