# Supplementary material for: Promoting positive parenting and mental wellbeing in Hong Kong Chinese parents: A pilot cluster randomised controlled trial
Source: PLoS One. 2022 Jul 20;17(7):e0270064. doi: 10.1371/journal.pone.0270064 (PMC9299310; doi:10.1371/journal.pone.0270064)
Supplement: Attachment — Submitted filename: renamed_ac5ab.docx [file pone.0270064.s005.docx]

**COMMENTS – Manuscript PONE-D-20-37785**

Title: “Promoting positive parenting and mental wellbeing in Hong Kong Chinese parents: a cluster randomised controlled trial”

**Important note: This review pertains only to ‘statistical aspects’ of the study and so** **‘clinical aspects’ [like medical importance, relevance of the study,** **‘clinical significance and implication(s)’ of the whole study, etc.] are to be evaluated [should be assessed] separately/independently. Further please note that any ‘statistical review’ is generally done under the assumption that (such) study specific methodological [as well as execution] issues are perfectly taken care of by the investigator(s). This review is not an exception to that and so does not cover clinical aspects {however, seldom comments are made only if those issues are intimately / scientifically related & intermingle with ‘statistical aspects’ of the study}. Agreed that ‘statistical methods’ are used as just tools here, however, they are vital part of methodology [and so should be given due importance].**

**COMMENTS:** In my opinion, sample size used is small because as per lines 36-38 [**Results:** Compared with the control group (n = 69), the intervention group (n = 75) showed greater positive changes in appreciation and enjoyment at 3-month with small effect sizes (d = 0.42 and 0.32, respectively)] the effect size is ‘small’ and small effect size study needs a larger sample size {refer to table-2, page-158 of: “A power primer” by J. Cohen in Psychological Bulletin, 1992, vol.:112, pp 155-159]. Considering that it is a ‘pilot’ (line 25), of course, sample size is not a big issue. [However, I very strongly feel that a word ‘pilot’ study should also appear in title.]

Since this is a [though pilot] cluster randomised controlled trial, statistical comparison of baseline characteristics [last ‘p-value’ column in Table 1] is not desirable. Please note

To provide a description of baseline characteristics is entirely reasonable (since it is clearly important in assessing to whom the results of the trial can be applied), however, it does not require the division of baseline characteristics by treatment groups (however, if done – alright). Statistical comparison of baseline characteristics is not desirable at all [because even if P-value turns out to be significant (while comparing baseline characteristics despite random allocation), it is, by definition, a false positive] as you then are supposed to be testing ‘randomization’ then, which in any single trial may not balance all baseline characteristics because ‘randomization’ is a sort of ‘insurance’ and not a guarantee scheme.

References:

1. Stuart J. Pocock, et al., ‘Subgroup analysis, covariate adjustment and baseline comparisons in clinical trial reporting: current practice and problems’, Statistics in medicine, 2002; 21:2917–2930 [Particularly page 2927]
2. Harrington D, et al., ‘New guidelines for statistical reporting in the journal’, N Engl J Med 2019;381:285-6

[Important message (indirectly/ultimately indicated) from these articles: Never do any comparison with respect to ‘baseline’ characteristics {by applying statistical significance test(s)}, when allocation is done randomly].

’P-values’ {last column} reported in Table 2 [The comparison between intervention group (n=75) and control group (n=69)] are supposed to have yielded by “Multilevel mixed-effects linear regression model” [as nothing has been given in the footnote]. It could have been simple to do it by non-parametric equivalent to unpaired ‘t’ test namely Mann-Whitney ‘U’ test on ‘change scores’ as though the measures/tools used are appropriate, most of them yield data that are in [at the most] ‘ordinal’ level of measurement [and not in ratio level of measurement for sure {as the score two times higher does not indicate presence of that parameter/phenomenon as double (for example, a Visual Analogue Scales VAS score or say ‘depression’ score)}]. Then application of suitable non-parametric test(s) is/are indicated/advisable [even if distribution may be ‘Gaussian’ (i.e. normal)]. Agreed that there is/are no non-parametric test(s)/technique(s) available to be used as alternative in all situation(s) [suitable / most desired/applicable], but should be used whenever/wherever they are available

Further, in my opinion, account given in ‘Focus groups and in-depth interviews’ section (lines 307-358) could be [and should be, I guess] reduced. Referring to lines 395-405 [Our study had several limitations…….] studies ‘quality’ becomes highly questionable. Even as a pilot study, in my considered opinion, this study does not contribute any new information.
