# Supplementary material for: Promoting positive parenting and mental wellbeing in Hong Kong Chinese parents: A pilot cluster randomised controlled trial
Source: PLoS One. 2022 Jul 20;17(7):e0270064. doi: 10.1371/journal.pone.0270064 (PMC9299310; doi:10.1371/journal.pone.0270064)
Supplement: Attachment — Submitted filename: Reponse to reviewers 20210808.docx [file pone.0270064.s006.docx]

**Authors' Responses to Comments**

**Reviewer 1’s comments**

C1. Important note: This review pertains only to ‘statistical aspects’ of the study and so ‘clinical aspects’ [like medical importance, relevance of the study, ‘clinical significance and implication(s)’ of the whole study, etc.] are to be evaluated [should be assessed] separately/independently. Further please note that any ‘statistical review’ is generally done under the assumption that (such) study specific methodological [as well as execution] issues are perfectly taken care of by the investigator(s). This review is not an exception to that and so does not cover clinical aspects {however, seldom comments are made only if those issues are intimately / scientifically related & intermingle with ‘statistical aspects’ of the study}. Agreed that ‘statistical methods’ are used as just tools here, however, they are vital part of methodology [and so should be given due importance].
    In my opinion, sample size used is small because as per lines 36-38 [Results: Compared with the control group (n = 69), the intervention group (n = 75) showed greater positive changes in appreciation and enjoyment at 3-month with small effect sizes (d = 0.42 and 0.32, respectively)] the effect size is ‘small’ and small effect size study needs a larger sample size {refer to table-2, page-158 of: “A power primer” by J. Cohen in Psychological Bulletin, 1992, vol.:112, pp 155-159]. Considering that it is a ‘pilot’ (line 25), of course, sample size is not a big issue. [However, I very strongly feel that a word ‘pilot’ study should also appear in title.]
R1. Thank you very much for reviewing our manuscript and your useful comments. We have now added “pilot” in title as suggested.
C2. Since this is a [though pilot] cluster randomised controlled trial, statistical comparison of baseline characteristics [last ‘p-value’ column in Table 1] is not desirable. Please note
    To provide a description of baseline characteristics is entirely reasonable (since it is clearly important in assessing to whom the results of the trial can be applied), however, it does not require the division of baseline characteristics by treatment groups (however, if done – alright). Statistical comparison of baseline characteristics is not desirable at all [because even if P-value turns out to be significant (while comparing baseline characteristics despite random allocation), it is, by definition, a false positive] as you then are supposed to be testing ‘randomization’ then, which in any single trial may not balance all baseline characteristics because ‘randomization’ is a sort of ‘insurance’ and not a guarantee scheme.
    References:
    1. Stuart J. Pocock, et al., ‘Subgroup analysis, covariate adjustment and baseline comparisons in clinical trial reporting: current practice and problems’, Statistics in medicine, 2002; 21:2917–2930 [Particularly page 2927]
    2. Harrington D, et al., ‘New guidelines for statistical reporting in the journal’, N Engl J Med 2019;381:285-6
    [Important message (indirectly/ultimately indicated) from these articles: Never do any comparison with respect to ‘baseline’ characteristics {by applying statistical significance test(s)}, when allocation is done randomly].
R2. We have now removed the comparison of baseline characteristics between groups and added the characteristics of the overall sample in **Table 1**. The unadjusted outcome analyses have now been included as **S4 Supplementary Table**.
C3. ’P-values’ {last column} reported in Table 2 [The comparison between intervention group (n=75) and control group (n=69)] are supposed to have yielded by “Multilevel mixed-effects linear regression model” [as nothing has been given in the footnote]. It could have been simple to do it by non-parametric equivalent to unpaired ‘t’ test namely Mann-Whitney ‘U’ test on ‘change scores’ as though the measures/tools used are appropriate, most of them yield data that are in [at the most] ‘ordinal’ level of measurement [and not in ratio level of measurement for sure {as the score two times higher does not indicate presence of that parameter/phenomenon as double (for example, a Visual Analogue Scales VAS score or say ‘depression’ score)}]. Then application of suitable non-parametric test(s) is/are indicated/advisable [even if distribution may be ‘Gaussian’ (i.e. normal)]. Agreed that there is/are no non-parametric test(s)/technique(s) available to be used as alternative in all situation(s) [suitable / most desired/applicable], but should be used whenever/wherever they are available
R3. We have added footnotes to Table 2: “p values were calculated using multilevel mixed-effects linear regression model.”. We used multilevel mixed-effects linear regression model as it contains both fixed effects and random effects. Individual participants in the same center were not independent. We used cluster RCT and the cluster effect should be adjusted for. Thus the Mann-Whitney ‘U’ test on ‘change scores’ may not be usable. We have now revised the statistical method section as follows to indicate the model fitting method, which is the default choice using xtmixed command in STATA.

**Line 277**: “Multilevel mixed-effects linear regression model (command XTMIXED) was used to calculate between-group mean differences (intervention vs. control) in the outcome changes, adjusting for clustering effect, significantly different demographics and baseline outcome variables, fitted via restricted maximum likelihood method.”

C4. Further, in my opinion, account given in ‘Focus groups and in-depth interviews’ section (lines 307-358) could be [and should be, I guess] reduced. Referring to lines 395-405 [Our study had several limitations…….] studies ‘quality’ becomes highly questionable. Even as a pilot study, in my considered opinion, this study does not contribute any new information.
R4. Focus groups and in-depth interviews are ways to triangulate the findings which have been used in many other trials^1^ to optimise the intervention and trial conduct, facilitate interpretation of the trial findings, help trialists to be sensitive to the human beings involved in trials. Our qualitative findings supported the effectiveness of our intervention in improving parents’ appreciation and enjoyment.

We are afraid that we do not agree with the reviewer’s comment that this study did not contribute to any new information. As stated in the introduction, only a few studies have used brief interventions to improve parenting especially in Chinese population. All of these studies adopted at least four group sessions of intervention [23-26]. Further shorter yet effective positive parenting preventive interventions have the potential to be adopted widely and sustainably if proved to be effective. Given the typical busy urban lifestyle in Hong Kong, brief interventions may encourage attendance and result in high adherence, especially for the general population who are self-perceived healthy. We think that our study has contributed to the evidence on designing very brief interventions for delivering positive parenting programmes.

**Reference**

1. O'Cathain A, Thomas KJ, Drabble SJ, Rudolph A, Hewison J. What can qualitative research do for randomised controlled trials? A systematic mapping review. BMJ open. 2013 May 1;3(6):e002889.

**Reviewer 2’s comments**
This paper investigated the efficacy of a brief positive parenting programme on or positive parenting (praise, appreciation, enjoyment) in a sample of 144 Hong Kong parents. The study had a mixed-method design, combining a randomized controlled trial (RCT) with group discussions. Quantitative measures were taken at baseline and again 1 month and 3 months post intervention. Group discussions with parents and individual discussions with providers were held post-intervention. Intervention effects were found for positive parenting and mental wellbeing. The promotion of positive parenting is of great importance, especially in a Hong Kong context, given the prevalence of “tiger parenting” within Hong Kong (and Chinese) communities. I commend the authors for undertaking this study and for drawing attention to the effects of positive parenting. Well done for recruiting 144 parents, for conducting implementation fidelity checks, and for controlling for confounders in the analysis.

C5. Introduction: The authors provide a rationale for the study, although the rationale for the actual intervention is not convincing to me. The introduction needs revision as there is some unclarity and a lack of nuance. For example:
    52: In Chinese culture, it is commonly thought that praising children breeds complacency and hinders learning
    54: “more than half of Hong Kong parents has used corporal punishment and 4.5% has maltreated their child physically”
    - I am confused by this sentence as, to me, corporal punishment is physical maltreatment. In my view, this sentence needs to be rephrased, looking carefully what the cited authors meant
R5. Thank you very much for reviewing our manuscript and your insightful comments and suggestions. We have now reorganized the introduction and rephrased the above two sentences as follows:

**Line 54**: “Western parents commonly use praise to boost children's self-esteem while Chinese parents tend to use less praise [3, 4].”

**Line 58**: “According to a report in 2006 including 1,662 Chinese parents with children aged 18 years or below, more than half of Hong Kong Chinese parents had ever used corporal punishment and 4.5% had maltreated their children physically with injuries [6].”

C6. The author defined corporal punishment as the use of physical force to inflict pain, but not injury, in disciplining children or controlling children's undesirable behaviors. Physical maltreatment refers to parents’ use of physical violence to inflict pain and injury on their children, which is more severe than that allowed by laws for disciplining children.

Tang CSK. Corporal punishment and physical maltreatment against children: A community study on Chinese parents in Hong Kong. Child Abuse Negl. 2006;30(8):893-907. doi: 10.1016/j.chiabu.2006.02.012. PMID: WOS:000240582000004.

- 56: The paper (5) that the authors cite is not focused on Chinese/ Hong Kong parents and is perhaps not the best reflection of Hong Kong parenting and child outcomes. Cultural differences exist in child outcomes following physical punishment. Some studies reported that African-American parents living in low SES areas use physical punishment as a strategy to keep their children safe, whereas European parents use physical punishment in anger. The child outcomes differ greatly between these cultures. The same may be the case for Chinese families.

R6. We agreed and have now removed the reference (5) (Gershoff ET., 2002) and its related sentence “Corporal punishment may achieve immediate compliance, but is an ineffective disciplinary practice associated with aggression, disrupted parent-child relationship and mental disorders used the studies”.

The 1st paragraph of the Introduction has been revised. Although the culture differences exist, the high prevalence of physical and psychological abuse, and the adverse outcomes, should not be neglected.

**Line 55**: “Chinese parents reported themselves as relatively low acceptance and warmth, but more hostile and neglecting than parents from other cultures [5]. Despite the culture differences across countries on the “training” or “controlling” concept [1], the high prevalence of child maltreatment is worrying.”

**Line 63**: “A meta-analysis of 22 studies in Chinese families showed that physical abuse was associated with adverse mental health outcomes [8].”

C7. Positive parenting protects against child behaviour problems. Indeed, studies have reported this, but the ones cited here (Shek et al, 2003 and Chronis et al 2007) are not the right ones to cite. It would be better to look for studies that have a longitudinal design, specifically looking at the effect of positive parenting and not in an ADHD sample (Chronis), as the authors’ study is not about ADHD.
R7. As we have reorganized the introduction part, the above two references and the related sentences have been removed. We kept two studies in Hong Kong and one of them was longitudinal study. Now we have also added the most recent findings about the associations between family support and adolescents’ life satisfaction.

**Line 70**: “Appreciation, warmth, affection and positive engagement predict children’s social competence and school achievement [11, 12],…”

**Line 66**: “Among different sources of social support, family support had the greatest positive impact on students’ life satisfaction and self-rated health [9, 10]. A recent study in Hong Kong reported that only 18% of young students were very satisfied with their current life, which was much lower than the international level of 36% [9].”

**Reference**

12. Chen X, Liu M, Li D. Parental warmth, control, and indulgence and their relations to adjustment in Chinese children: a longitudinal study. J Fam Psychol. 2000;14(3):401-19. doi: 10.1037//0893-3200.14.3.401.

C8. 68: “typical problem and treatment oriented parenting programmes have limited impact at the population level.” I don’t think this is true and the cited paper doesn’t claim this.
R8. We have rephrased the sentence as “Population-based approach is recommended to extend the impact of evidence-based interventions at the population level [18, 19].”

**References**

18. Prinz RJ, Sanders MR. Adopting a population-level approach to parenting and family support interventions. Clin Psychol Rev. 2007;27(6):739-49. doi: 10.1016/j.cpr.2007.01.005.

19.	Murray RM, Cannon M. Public health psychiatry: an idea whose time has come. World Psychiatry. 2021;20(2):222-3. doi: 10.1002/wps.20868.

C9. 75: I don’t understand the proposed need for shorter positive parenting programmes. Shorter than Triple P? Triple P has many different intervention levels, including very brief ones. Incredible Years is rather long, but the authors don’t refer to this programme
R9. We have revised the paragraph as below.

**Line 92**: “Brief and inexpensive interventions, if effective, can be adopted widely and sustainably [23]. Given the typical busy urban lifestyle in Hong Kong, brief interventions may encourage attendance and result in high adherence, especially for the general population who are self-perceived healthy. Only a few studies using less intensive positive parenting interventions were conducted among Chinese parents [23-26]. All of these studies adopted at least four group sessions of intervention [23-26]. Further shorter yet effective positive parenting preventive interventions can be designed and assessed, but evidence is limited.”

C10. 80: I assume to Joyful Parenting Pilot Project aimed to promote positive parenting behaviours amongst parents (not just any adult). Did this project involve several interventions? I would like to learn a bit more about the actual intervention. How do fun family games lead to increased praise?
R10. Family games is to engage the parents into the activities and parents learn how to give praise and show appreciation in the process. We have now added more details about the intervention as follows

**Line 168**: “Three different kinds of simple family games were led by the social workers to let the parents experience how to give praise. The parents could observe and think about how to give praise or show appreciation in the process. They tried to praise each other through playing the games cooperatively. The principles of praise were emphasised: give detailed and specific praise by using more adjective words (specific praise), praise for improvements made rather than focusing on the achievements they made (give outcome praise in the right way: e.g. The parents had a high expectation on children’s academic performance, but children’s improvement from grade C to B was still worth praising), praise for their efforts made in the process even if they fail (process praise), have a consistent and reasonable standard, and be sincere.”

**Line 186**: “Lego games and role-playing games were organised in group format. Same as the first talk, these games were designed to remind the parents to think about which part of the games, who, and what behaviours they want to give praise for. The parents were provided with the materials of the Lego and were encouraged to play together with their children at home and show appreciation in the process.”

C11. 87: This sentence implies that only qualitative data were collected.
R11. We have now revised the sentence as below.

**Line 111**: “Qualitative data were also collected through focus groups in parents and in-depth interviews in community service providers.”

C12. Methods: How were demographics obtained? Why were participants randomised by cluster? What were the inclusion and exclusion criteria? How were parents within the centres recruited?
R12. We have included the reasons of choosing cRCT, the inclusion and exclusion criteria as follows. The centres did recruitment using public posters to recruit community residents as many as possible rather than focused on their existing clients.

**Line 120**: “For each integrated family service centre of the HKFWS, some clients use the services frequently and many of them may know each other well. Therefore, cluster design (assigning the participants recruited by the same centre to the same group) was adopted to avoid the potential contamination between the intervention and control groups.”

**Line 125**: “Parents aged 18 to 59 years were recruited by the four service centres through leaflets and a public poster in the centres. Parents who were able to speak Chinese and complete questionnaires were eligible. Those who cannot read Chinese or were suffering from severe mental illness were excluded. Grandparents or other types of caregivers were not included.”

C13. 99: “Family members (could be children or adolescents) of the participating parent were also invited to join a family gathering activity after completion of the interventions” What does this mean? Is this essential to understanding the procedure (i.e. replicating the study)?
R13. Family gathering activity was not a part of the intervention as the final follow-up assessment had been completed before that. It only served as a kind of incentive. We have revised it as follows. Replicating the study does not require this part but other types of incentives may be provided to the participants.

**Line 144**: “We measured all the outcomes at baseline (before the first session of intervention), conducted the first follow-up assessment at 1 month (before the second session of intervention) and the second/final follow-up assessment at 3 months. A family gathering activity was provided to the participants after the final outcome assessment, which was used to encourage the participation and thank the participants for their completion of questionnaires.”

C14. 102: “The study protocol (dated 1 June 2017) was fixed before the ethical approval and enrollment of participants.” What does this mean? Again, is this essential for the study to be replicated?
     Ethical approval:
     Was assent obtained from underaged children over 7 years?

Clinical trial registry data is mixed up with ethical approval data. Please separate these.

R14. We have now deleted the sentence “The study protocol (dated 1 June 2017) was fixed …” Consent was obtained from all children. We have separated the ethical approval description with the trial registry as follows:

**Line 131**: “Ethical approval was obtained from the Institutional Review Board of the University of Hong Kong/Hospital Authority Hong Kong West Cluster (reference number: UW17-240, dated 5 July 2017). Written informed consent was obtained in adult participants and parents provided written consent for children under the age of 18. The study was registered (NCT03282071, S1 Study Protocol). We reported the results following CONSORT guideline (S2 CONSORT 2010 checklist).”

C15. Procedure:
     The authors state that randomization happened via numbers generated by a computer, but continue by stating how group allocation occurred using envelopes.

The intervention procedure doesn’t make sense to me. Baseline levels were taken, measures were repeated after the first talk and again after the second talk, after which family activities took place. A more common method would be to measure baseline levels, provide the intervention to the intervention group, repeat the measures post-intervention and again at follow-up. After all assessments are done, the control group receives the intervention.

R15. We generated random sequence using computer and use sealed envelopes containing random sequence for random allocation (concealment) following the standard SNOSE procedure.

We have now revised the description of assessment period to make the process more clear (please also refer to **R13**). The first follow-up was to capture the effect of the first talk after one-month. We would like to know the effect of the first session because if the 1-month evaluation shows significant positive changes, a single session of intervention may also be applicable in future trials. If we conducted the second assessment immediately after the interventions (after the second talk, post-intervention), the effect might be largely due to the second talk and we could not know the effect of the first talk clearly.

C16. Intervention:
     Describe what “Sharing, Mind and Enjoyment” and “knowledge of Mixed Anxiety and Depressive Disorder (MADD)” mean instead of just using names. Also, I would suggest rewriting this section to separate the measures (such as MADD) from the actual intervention. Be really precise in describing the intervention as this is a new intervention and readers will not have heard of it. What does interactive talk entail? It sounds like a lecture provided to parents.
     Did control group parent take part in the family gathering activity? Was this event used as incentive?
R16. Response 12:

We have now described more about the SME and separated the MADD part.

**Line 152**: “Sharing, Mind and Enjoyment (SME) was used as the slogan of the Joyful@HK campaign. Sharing connects family and friends, and supports those in need. Mind entails keeping an open mind and be positive and optimistic. Enjoyment is about engaging in enjoyable activities to maximise one’s potential and achieve satisfaction. As the SME concept was broad, the specific techniques of the intervention in this project were praise (positive sharing), appreciation (positive mind) and family games to promote enjoyment.”

**Line 162**: “In the first interactive talk, the instructors first introduced the symptoms of MADD to the participants briefly and the ways to seek help for emotional disturbances, given that the most common mental disorder in Hong Kong adults was Mixed Anxiety and Depressive Disorder (MADD) (6.9%) [29] and parents were commonly stressful. Then the instructors brought the importance of positive mind and positive discipline to build a positive environment and promote positive parent-child relationship.”

For the comments about the family gathering activity, please refer to **R13**.

C17. Outcome measures: It is generally better to include measures that have been validated and that are reliable, especially for primary outcome measures. With self-developed measures, you don’t know if you are measuring what you want to measure. I have strong doubts about the validity and reliability of the primary outcome measures. For the next study, I would suggesting looking at existing literature to review what kind of measures can be used to measure your outcomes of interest. This also enables comparison between studies.
R17. We agree with the reviewer about using valid and reliable measurements although one or two-item measures could show good validity.^1^ The measurements could be improved in our future studies. We have now added the correlations of these measurements with the valid scales used in our study. However, as our study did not include a second validated scale about praise or positive parenting, the validity of our primary outcome measurement was uncertain. We have also stated this as our limitation.

**Line 193**: “We developed the outcome and impact-oriented questionnaires to assess the changes in the participants. Praise, appreciation and enjoyment were consistent with our delivered content of the intervention (sharing, mind and enjoyment)…. Significant correlations were found between praise, appreciation, enjoyment and subjective happiness, wellbeing (r = 0.19-0.43, all p < 0.05), indicating that these measurements have acceptable validity in estimating better wellbeing and higher level of subjective happiness.”

**Line 458**: “First, because validated questionnaires were not available and many existing questionnaires were too long, we developed the outcome and impact-oriented questionnaires to assess the changes in the participants. The measurements showed acceptable to good congeneric reliability. Future studies should test the validity of the praise measurements.”

**Reference:**

1. Jensen MP, Keefe FJ, Lefebvre JC, Romano JM, Turner JA. One-and two-item measures of pain beliefs and coping strategies. Pain. 2003 Aug 1;104(3):453-69.
C18. In the abstract it is unclear what the purpose was of the focus group discussions and the interviews. The methods explains the purpose of the group discussions, but not of the interviews.
     Also (re information in the abstract), it is more informative to know how many parents took part in a focus group discussion. It is less informative to report on the number of group discussions, as two parents can be considered a group, as well as 10. A group of 10 people will likely provide more information than a group pf two people.
     Analysis of the qualitative data should not be included in the statistical analysis section.
R18. We have now included more information about the focus group and in-depth interviews.

**Line 35:** “After the completion of all assessments, five focus group discussions with the parents and four individual in-depth interviews with community service providers were conducted to explore their experiences.”

**Line 259**: “The focus group discussion was mainly to explore parents’ satisfaction with the contents, subjective changes and suggestions for future programmes. The in-depth interviews were to explore community partners’ perceptions on the usefulness, difficulties in implementation and suggestions.”

**Line 266**: “Five focus group discussions of parents were conducted, with an average of 9 parents in each group (7-12 parents per group, 34 women and 11 men).”

We have now changed the subtitle of “Statistical analysis” to “Data analysis”.

C19. Results
     I appreciate the distinction the authors made between primary and secondary outcomes. Table one needs revision to include outcomes of the t-tests/ chi-square tests (please check APA guidelines for required content). How was the effect size calculated? Why is the effect size not included in Table 2? SD is also not reported.
     I am not convinced of the results, mainly due to the self-developed primary outcome measures and the timing of the second set of assessments (during the intervention). Presentation of the results is sometimes unclear, e.g.,
     263: “parents gave praise in 7.13 (SD 5.21), 6.69 (5.79), 6.63 (5.83) and 4.48 (5.84) days from the first to the fourth week” According to the methods section, the maximum score is 84. The scores presented in the results appear rather low. Also, the results should present the intervention effects, not mean scores.
R19. Response 15:

We have now removed the p-values from Table 1 according to the statistical comments from Reviewer 1. Effect size was reported in Table 2 as Cohen’s d. We also have included SD of the mean values.

About the primary outcome, please refer to **R17**. Furthermore, most secondary outcomes were validated scales and had shown significant positive changes when compared the intervention group with the control group. The effect of the intervention were also supported by subjective changes and qualitative findings.

About the timing of the measurement, please refer to **R13**.

We have now included more information about the worksheets. The unit of Praise (days) is the number of three types of praise per week, ranging from 0 to 21. This was only reported by the intervention group to record the frequency of giving praise after the first talk. Therefore, no intervention effects could be reported.

**Line 178**: “Simple worksheets were assigned to the parents to record their practice of three types of praise (specific praise, process praise and outcome praise) and family games they arranged in the following four weeks, resulting a maximum of 21 days of praise and unlimited times of family games in each week.”

C20. I feel as if the thematic analysis could have yielded more in-depth results. If more in-depth data is available it may be interesting to present the group discussions and individual interviews in a separate paper.
R20. Thanks for the suggestion. We thought it would be more appropriate to keep the results together with the quantitative data for better explaining the effect. As the present paper is a pilot, qualitative data could help readers learn more about the participants’ perspectives and experiences.
C21. Discussion
     With the summary of the essential findings, it is unclear if the findings refer to the 1-month or the 3-month assessments. The authors state they expected small effect sizes, however, this was not stated in the previous sections. Why did the authors expect small effect sizes? The qualitative findings are not discussed (or I may have missed this part).
     The potential theoretical and practical implications have not been identified. Apart from the suggestion to have a larger sample size, the authors do not provide suggestions for future research.
R21. We have now added the time-points accordingly.

We have now removed the sentence regarding the “expected small effect sizes”.

The discussion about the qualitative findings were also included, such as,

**Line 441**: “Some parents also reported difficulties in temper control, especially when they were not satisfied with their children’s performance in schools.”

**Line 433**: “We did not observe significant changes in praise, according to the quantitative data. However, more than 95% parents reported that they gave more praise and appreciation to their children. The qualitative data have also shown more praise in some parents.”

C22. Additional comments:
     As an English as second language speaker myself, I appreciate how difficult and illogical the English language can be. I would suggest to seek editorial assistance from a native English speaker. There are quite a few improvements to be made throughout the paper, e.g.
     28: Two talks were delivered, including praise and appreciation skills, and enjoyable family games  this implies that “praise”, “appreciation skills”, and “enjoyable family games” are talks, while in effect these are skills taught or discussed during the talks.
     30: at baseline, 1 month, and 3 months. Note: singular (and hyphen) is used in other cases, e.g. 3-month follow-up, at 3 months
     31: when expressing comparisons (e.g. greater), you need to include what it is compared to, e.g. we expected greater improvement reported by intervention group parents as compared to those in the control group
     39: small effect sizes (plural)
     83: Praise is always singular, as is content (127)
     84: easy-to-catch family games? What are those? Simple games that are easy to learn?
     95 “under the Hong Kong Family Welfare Society”
     99: “Family members (including children)”
R22. Thanks for the suggestions.

We have done proofreading of the text throughout.

**Line 28**: “The intervention included two 2-hour interactive talks (4 hours in total). The contents included praise, appreciation skills and enjoyable family games.”

The time points have been checked and revised throughout the text.

Singular and plurals were checked. The other grammar issues have been revised accordingly.

We have removed the “easy-to-catch family games” from the text.

**Line 120 and 125**: “of the HKFWS”

C23. Some of the references are a bit old. Some more Chinese/Hong Kong studies (Triple P):
     Chan S, Leung C, Sanders M (2016) A randomized controlled trial comparing the effects of directive and non-directive parenting programs as a universal prevention program. J Child Serv 11: 38-53
     Guo M, Morawska A, Sanders MR (2016) A randomized controlled trial of Group Triple P with Chinese parents in mainland China. Behav Modif 40: 825-851.
R23. Thanks for the recommended papers. We have added them in the introduction.

**Line 95**: “Only a few studies using less intensive positive parenting interventions were conducted among Chinese parents [23-26]. All of these studies adopted at least four group sessions of intervention [23-26].”

**Reviewer 3’s comments**

C24. Abstract: The abstract would benefit from being more concise, particularly under methods. The hypotheses shouldn’t be stated under the methods. The authors should include information on the sample (e.g., parents of children of what age? Were this parents with concerns for their children or general public?) When did the study take place and duration? Were the focus groups conducted after or before the interventions and what were the focus groups for? The concluding statement seems to be exaggerated given the small effect size and short follow up duration. What is the impact for such a trial?
R24. Thank you very much for the suggestions.
     We have revised the abstract and added the “school age children”, “conducted in 2017”, “After the completion of all assessments, five focus group discussions with the parents and…”, “…has shown preliminary effectiveness in…”.

As stated in the introduction, only a few studies have used brief interventions to improve parenting especially in Chinese population. All of these studies adopted at least four group sessions of intervention [23-26]. Further shorter yet effective positive parenting preventive interventions have the potential to be adopted widely and sustainably if proved to be effective. Given the typical busy urban lifestyle in Hong Kong, brief interventions may encourage attendance and result in high adherence, especially for the general population who are self-perceived healthy. Our study has contributed to the evidence on designing very brief interventions for delivering positive parenting.

C25. Introduction: The introduction presents Chinese parenting style. It is not clear to the reader what age group of children the authors are referring to. The introduction currently is weak and does not present a good rationale for why brief parenting programmes are needed in Hong Kong. More context should be given. The objective is to promote positive parenting practices and mental wellbeing in parents, yet no discussion is given on why parent mental wellbeing is important and how this link to children wellbeing.
R25. We have now revised the whole section of Introduction and reorganized the rationale.

**Line 92**: “Brief and inexpensive interventions, if effective, can be adopted widely and sustainably [23]. Given the typical busy urban lifestyle in Hong Kong, brief interventions may encourage attendance and result in high adherence, especially for the general population who are self-perceived healthy. Only a few studies using less intensive positive parenting interventions were conducted among Chinese parents [23-26]. All of these studies adopted at least four group sessions of intervention [23-26]. Further shorter yet effective positive parenting preventive interventions can be designed and assessed, but evidence is limited.”

**Line 79**: “Common child-rearing challenges are experienced by a substantial proportion of parents. Low household income and education [15], low family functioning [16], children’s behavioural problems [15] and high expectation towards children [17] were all the sources of parental stress. Culturally, Chinese parents place high expectations on children’s academic performances, which may make them experience more parental stress. Furthermore, the vast majority of parents may not seek out services through mental health providers, either for themselves or their children. The limited resources of services and potential stigma may be barriers to access to consultation. Population-based approach is recommended to extend the impact of evidence-based interventions at the population level [18, 19]. A successful example is the Triple P model, which is a multi-level, preventive parenting and family support strategy to enhance family protective factors and reduce risk factors associated with maltreatment [20, 21]. To enhance parental competence and parenting practices and to reduce the risk of child maltreatment in the community, the public health or universal approach is needed [22].”

C26. Line 52: Is this a belief or is there evidence to support such claim?
R26. There is evidence to support such claim.

**Line 51**: ““Guan” (training, control or governance) has a very positive connotation in Chinese culture, meaning to govern and train but also to show care and love [1].”

C27. Line 54 the author cited that more than half of Hong Kong parents has used corporal punishment. This statement requires further elaboration otherwise is out of context. The authors should state whether this data was from a survey and the sample size of the survey.
R27. We have revised the text as follows.

**Line 56**: “Despite the culture differences across countries on the “training” or “controlling” concept [1], the high prevalence of child maltreatment is worrying. According to a report in 2006 including 1,662 Chinese parents with children aged 18 years or below, more than half of Hong Kong Chinese parents had ever used corporal punishment and 4.5% had maltreated their children physically with injuries [6]. According to a latest report in 2019 on Hong Kong children attending Grade 1 to 3 (aged 6-10 years), the past year prevalence of minor physical abuse, severe abuse, psychological abuse, and neglect were 63.9%, 23.4%, 84.1%, and 23.2%, respectively [7].”

C28. Line 60: what does the author mean by children’s social achievement?
R28. Sorry for the misunderstanding caused. We have revised the text as follows.

**Line 71**: “children’s social competence and school achievement”

C29. Line 61-63: Not sure what the author mean in this sentence. What does the author mean by reward? Reward does not always necessarily lead to positive behaviour but can increase undesired behaviours in children.
R29. We have rephrased the sentence as below.

**Line 72**: “Chinese parents showing warmth and reward to children is more of getting children’s compliance to parental expectation than encouraging children’s independence and competence [13].”

C30. Line 68-75: Does the author mean targeted/selected parenting program? The authors should explain the public health model and why universal approaches are needed for prevention. Existing literatures should be drawn and expand on. The authors should also expand on the few studies that were conducted on Chinese parents. A stronger rationale for brief parenting programmes is needed.
R30. We have revised the text as below.

**Line 86**: “Population-based approach is recommended to extend the impact of evidence-based interventions at the population level [18, 19]. A successful example is the Triple P model, which is a multi-level, preventive parenting and family support strategy to enhance family protective factors and reduce risk factors associated with maltreatment [20, 21]. To enhance parental competence and parenting practices and to reduce the risk of child maltreatment in the community, the public health or universal approach is needed [22].

Brief and inexpensive interventions, if effective, can be adopted widely and sustainably [23]. Given the typical busy urban lifestyle in Hong Kong, brief interventions may encourage attendance and result in high adherence, especially for the general population who are self-perceived healthy. Only a few studies using less intensive positive parenting interventions were conducted among Chinese parents [23-26]. All of these studies adopted at least four group sessions of intervention [23-26]. Further shorter yet effective positive parenting preventive interventions can be designed and assessed, but evidence is limited.”

C31. Line 80: What do the authors mean by pilot engagement trial?
R31. We have rephrased the sentence as below.

**Line 101**: “The present trial, Joyful Parenting Pilot Project, was one of the community-based participatory research (CBPR) projects under the campaign to promote mental health among adults in Hong Kong. CBPR is an approach to health and environmental research meant to increase the value of studies for both researchers and the community being studied [28].”

C32. Method: Family service centres were engaged to recruit. Were these parents already clients from the service centre? The authors stated that the family centres provide comprehensive and extensive professional support, does this mean that their service clients were those at risk or a high risk? A definition should be given re parents. Did the authors only recruited parents? What about caregivers? The inclusion and exclusion criteria should be clearly stated. Given the brief nature of the intervention, did the research team exclude families that were at risk? If so, what were used to screen families? Line 108: What about child assent? If the children did not want to participate, does that mean the family is excluded? Consort diagram should be included. More information on recruitment should also be given.
R32. We have included more about the inclusion criteria as follows. As the recruitment was done through public posters, all the community residents could participate. Those with severe mental illness were excluded. According to the recruitment flow, only 8 parents who were over 59 years old were excluded. Consort diagram was included as Figure 1. The children did not participate into the two interactive talks, so they did not affect their parents’ participation.

**Line 125**: “Parents aged 18 to 59 years were recruited by the four service centres through leaflets and a public poster in the centres. Parents who were able to speak Chinese and complete questionnaires were eligible. Those who cannot read Chinese or were suffering from severe mental illness were excluded. Grandparents or other types of caregivers were not included.”

C33. Line 119: why were the assessment completed before the intervention were completed? If the 3 month assessment was conducted before the family gathering activities, how did the authors measure the effect of the family activities? The authors can consider using a diagram to show the intervention and schedule of assessments. A table can also be used to explain the content of the intervention. What was the reason for the one month gap between the two talks? Why lego?
R33. The 3-month assessment was conducted before the family gathering activities. Therefore, we only measured the effect of two interactive talks. The effect of the family gathering activities was not measured as a part of the intervention effect, because we assume the post-intervention effect of the interventions will be mixed by the free lunch gathering. Furthermore, it is not easy to gather all the parents to complete the questionnaire without any activities being provided.

The one-month gap between the two talks was to let the parents have time to practice the learned skills. Also, the community partners need time to arrange another intervention sessions for the parents.

We have now also included more details about the interventions and a table to show the intervention and schedule of assessments (S3 Programme Rundown).

The games were unnecessary to be fixed on one specific type. They could be other types of games as long as the local community practitioners thought they were useful and could engage the participants into the games and let them practice the learned skills.

**Line 168**: “Three different kinds of simple family games were led by the social workers to let the parents experience how to give praise. The parents could observe and think about how to give praise or show appreciation in the process. They tried to praise each other through playing the games cooperatively. The principles of praise were emphasised: give detailed and specific praise by using more adjective words (specific praise), praise for improvements made rather than focusing on the achievements they made (give outcome praise in the right way: e.g. The parents had a high expectation on children’s academic performance, but children’s improvement from grade C to B was still worth praising), praise for their efforts made in the process even if they fail (process praise), have a consistent and reasonable standard, and be sincere.”

**Line 186**: “Lego games and role-playing games were organised in group format. Same as the first talk, these games were designed to remind the parents to think about which part of the games, which person, and what behaviours they want to give praise for.”

C34. Line 149: What was the validity of the scale for measuring praise. How confident are the authors in regard to recall bias? The reliability of the enjoyment scale appear just acceptable.
R34. The reliability of our primary outcomes was acceptable (0.88, 0.89, 0.62 for praise, appreciation and enjoyment, respectively). We included this as a major limitation in the discussion but have included the correlation with the other validated scales for reference. Please see the revisions as follows.

**Line 205**: “Significant correlations were found between praise, appreciation, enjoyment and subjective happiness, wellbeing (r = 0.19-0.43, all p < 0.05), indicating that these measurements have acceptable validity in estimating better wellbeing and higher level of subjective happiness.”
**Line 458**: “First, because validated questionnaires were not available and many existing questionnaires were too long, we developed the outcome and impact-oriented questionnaires to assess the changes in the participants. The measurements showed acceptable to good congeneric reliability. But the validity of the measurement should be established in future studies by testing its correlations with other measurements of positive parenting.”

C35. Results: Line 299 There were no mention that children were required to complete assessments, this should be stated clearly in the methods section. Was specific questionnaires used to collect response from children? How reliable are data collected from a 4 year old?
R35. We have now included below information in the methods section:

**Line 250**: “At 3 months, the children were invited to complete a short questionnaire by themselves or with the help of their parents. Their self-perceived praise, appreciation and enjoyment were asked: “how many days did your parents praise for your efforts in words or actions / praise for your strengths in words or actions / encourage you in words or actions / observe your strengths carefully / understand you in a positive way / enjoy the time with you / hold outdoor activities with you?”. Questions about personal health and happiness, family health, happiness and harmony were also asked, which were same as the parent’s version.”

C36. Discussion: The authors state that the primary outcome is positive parenting practices, however the self-developed scales does not seem to measure parenting practices. For example, a measure on enjoyment. Can the author justify why these scales were chosen or used?

It was unclear in the methods section that there were worksheet available for parents. This should be stated in the methods.

The authors didn’t find significant changes in praise, and suggested that parents beliefs in the usefulness of criticism may be difficult to change in short-term. What are the implications of such findings? Perhaps modification to the content of how praise are taught?

And how can the authors reduce the use of criticism in parents?

Line 384: How does appreciation and enjoyment link to quality of communication?

There are very few male participants, any thoughts on how this can be improved?

R36. Now we have now added the reasons of developing these scales.

**Line 193:** “*We developed the outcome and impact-oriented questionnaires to assess the changes in the participants. Praise, appreciation and enjoyment were consistent with our delivered content of the intervention.”*

We have now added the information about the worksheets in the method as below.

**Line 178:** “Simple worksheets were assigned to the parents to record their practice of three types of praise (specific praise, process praise and outcome praise) and family games they arranged in the following four weeks, resulting a maximum of 21 days of praise and unlimited times of family games in each week.”

We explained the reasons of the non-significant changes in praise in the discussion. The timing of the measurement might have affected the quantitative results. If we asked their behaviours across one month, the effect might be larger. But we asked their praise behaviours “in the past seven days”, which might have decreased after several weeks of practice. We think the self-reported subjective changes and qualitative data could also be the evidences of the intervention effect.

**Line 434**: “However, more than 95% parents reported that they expressed more praise and appreciation to their children. The qualitative data have also shown more praise in some parents. The small sample size of the pilot study might have led to the non-significant increase of parents’ praise behaviour. Another possible explanation is that the practice of praise and family games has just decreased at the 1 month evaluation point. According to the collected worksheets of home practice, the 4th week practice of praise and family games reduced significantly, while our one month outcome evaluation just documented that period.”

We did not measure criticism in the current study, but it would be interesting to do so in the future study. We have to admit that “more praise” does not mean “less criticism”. But positive parenting programs have the potential to prevent or reduce physical and psychological abuse.

**Line 444**: “We did not measure criticism or abuse in the present study but it would be useful to include the measurements in future study, to evaluate whether such programs could prevent or reduce children maltreatment.”

We have revised the “quality of communication” to “mindset”.

**Line 447**: “it is possible that the intervention has changed the mindset of the parents as evidenced by the higher level of appreciation and enjoyment at 3 months.”

We have added a little bit in the discussion about the recruitment of fathers.

**Line 468**: “Given the difficulties in recruiting fathers, and that mothers may have a stronger influence on the children rearing in Hong Kong, targeting mothers seems to be an effective strategy.”

C37. There are grammatical errors and typos throughout the manuscript, the manuscript would benefit from careful proof reading.
R37. We have proofread the manuscript thoroughly and made necessary corrections.
C38. The author stated that data cannot be shared due to consent reasons. Data that are deidentified should be made available upon request for example for inclusion into meta-analyses. Given journal policy for data sharing and availability, the authors should consider the inclusion of such statement in consent forms and PIS in future studies.
R38. Now we have stated this in the data availability statement “Data without personal information is available upon reasonable requests”. We will include such data availability statement in future studies.

- The End -
