# Supplementary material for: Promoting positive parenting and mental wellbeing in Hong Kong Chinese parents: A pilot cluster randomised controlled trial
Source: PLoS One. 2022 Jul 20;17(7):e0270064. doi: 10.1371/journal.pone.0270064 (PMC9299310; doi:10.1371/journal.pone.0270064)
Supplement: Attachment — Submitted filename: renamed_95586.docx [file pone.0270064.s007.docx]

**COMMENTS – Manuscript PONE-D-20-37785R1**

Title: “Promoting positive parenting and mental wellbeing in Hong Kong Chinese parents: a pilot cluster randomised controlled trial”

**COMMENTS:** I noted that, ABSTRACT is well drafted but assay type. Please note that it is preferable to divide the ABSTRACT with small sections like ‘Objective(s)’, ‘Methods’, ‘Results’, ‘Conclusions’, etc. which is an accepted practice of most of the good/standard journals [including this one]. If I remember correctly, it was divided in small sections earlier. May please consider (again).

Thank you very much for adding a word “pilot” in title and removing ‘p-value’ column in Table 1 [i.e. statistical comparison of baseline Characteristics] as suggested {both actions highly appreciated}. I completely agree that “the cluster effect should be adjusted for and thus the Mann-Whitney ‘U’ test on ‘change scores’ may not be usable”, but wanted to indicate that direct / head-to-head comparison between groups is desirable [that does not mean that use of “Multilevel mixed-effects linear regression model” which definitely adjust for clustering effect is wrong but note that this technique is not originally developed for comparison of between-group mean differences. In this context [authors may already know the reference], here is a good reference: Donner Allen and Klar Neil. `Design and Analysis of Cluster Randomization Trial in Health Research’, Oxford University Press Inc., New York, 2000.

The fact that ‘though the measures/tools used here are appropriate, most of them yield data that are in ‘ordinal’ level of measurement [and not in ratio level of measurement for sure {as the score two times higher does not indicate presence of that parameter/phenomenon as double (for example, a Visual Analogue Scales VAS score or say ‘depression’ score)}].

I certainly know the vital importance of ‘Focus groups and in-depth interviews’ in research but I only suggested to reduce account given in section.

Ultimately, in my considered opinion now, ‘let the respected editor decide the future course’. I do not have any specific recommendation.
