# Supplementary material for: Promoting positive parenting and mental wellbeing in Hong Kong Chinese parents: A pilot cluster randomised controlled trial
Source: PLoS One. 2022 Jul 20;17(7):e0270064. doi: 10.1371/journal.pone.0270064 (PMC9299310; doi:10.1371/journal.pone.0270064)
Supplement: Attachment — Submitted filename: Response to Reviewers.docx [file pone.0270064.s008.docx]

**Authors' Responses to Comments**

**Reviewer 1’s comments**

**C1.** I noted that, ABSTRACT is well drafted but assay type. Please note that it is preferable to divide the ABSTRACT with small sections like ‘Objective(s)’, ‘Methods’, ‘Results’, ‘Conclusions’, etc. which is an accepted practice of most of the good/standard journals [including this one]. If I remember correctly, it was divided in small sections earlier. May please consider (again).

**R1.** Thanks very much for your comments and suggestions. Now we have divided the abstract into small sections.

**C1.** Thank you very much for adding a word “pilot” in title and removing ‘p-value’ column in Table 1 [i.e. statistical comparison of baseline Characteristics] as suggested {both actions highly appreciated}. I completely agree that “the cluster effect should be adjusted for and thus the Mann-Whitney ‘U’ test on ‘change scores’ may not be usable”, but wanted to indicate that direct / head-to-head comparison between groups is desirable [that does not mean that use of “Multilevel mixed-effects linear regression model” which definitely adjust for clustering effect is wrong but note that this technique is not originally developed for comparison of between-group mean differences. In this context [authors may already know the reference], here is a good reference: Donner Allen and Klar Neil. `Design and Analysis of Cluster Randomization Trial in Health Research’, Oxford University Press Inc., New York, 2000.

**R2.** Thanks for the recommended reference, which suggested conducting an individual level analysis using mixed effects regression. Yet the statistical inferences should be constructed based on large sample theory. However, as a pilot trial, only four family centres were involved in our study. This limitation has been included in the discussion. XTMIXED is used to fit linear mixed models and the overall error distribution of the linear mixed model is assumed to be Gaussian (i.e. normal). The distribution of our primary and secondary outcomes was normal or close to normal according to the histograms, so we adopted multilevel mixed-effects linear regression model to calculate between-group mean differences.

**C3.** The fact that ‘though the measures/tools used here are appropriate, most of them yield data that are in ‘ordinal’ level of measurement [and not in ratio level of measurement for sure {as the score two times higher does not indicate presence of that parameter/phenomenon as double (for example, a Visual Analogue Scales VAS score or say ‘depression’ score)}].

**R3.** We agree with the reviewer on the limitation of the measurement. Therefore, we reported Cohen’s d, a standardized mean difference, to measure the between group difference.

**C4.** I certainly know the vital importance of ‘Focus groups and in-depth interviews’ in research but I only suggested to reduce account given in section. Ultimately, in my considered opinion now, ‘let the respected editor decide the future course’. I do not have any specific recommendation.

**R4.** We have now shortened the descriptions while keeping the sections. We will certainly consider reporting the findings separately in the future definitive RCT with a more solid qualitative study.

**Reviewer 2’s comments**

**C1.** The authors put in a lot of effort to take on board reviewers’ suggestions. However, there are still outstanding issues that need to be addressed, plus some new ones due to the changes in the introduction. There are still quite a few English grammar mistakes. E.g.,

a. line 74: “meaning to govern and train but also to show care and love”, line 78 “reported themselves as low acceptance”,

b. line 83 “a latest report”. Please make use of a proficient English speaker for the next version.

c. Line 111: “Chinese parents showing warmth and reward to children is more of getting children’s”

d. Line 173: “Those who cannot read Chinese or were suffering from “
My comments relate to the revision with the track and changes.

**R1.** Thanks for the comments and very specific suggestions. The paper has been proofread by a proficient English speaker. We have revised the sentences as below. The line numbers relate to the new clean version.

a. Line 55: *“meaning governing and training but also showing care and love”*

Line 59: *“A lower degree of acceptance and warmth were reported in Chinese parents”*

b. Line 64: *“Another report in 2019”*

c. We have deleted the sentence “Chinese parents showing warmth and reward to children is more of getting children’s”.

d. Line 130: *“Those who could not read Chinese or had severe mental illness were excluded.”*

**C2.** Abstract:

The abstract could benefit from some reorganising.

a. It is helpful to stick to the common “intro, methods, results, discussion, conclusion”. Be succinct and only state the essential.

b. Line 35: Please add children’s mean age.

c. Line 38: I am unclear what the difference may be between praise and appreciation skills.

d. Line 39: remove the comment on the control group not receiving an intervention during the study. This is how control groups work. Instead, you could state, “the control group participants were offered the intervention after all data were collected”

e. I would advise to make a distinction between primary outcomes and secondary outcomes in the results reported in the abstract as well.

**R2.** Thanks for the comments on the Abstract.

a. Now we have divided the abstract into small sections.

b. We have added children’s mean age.

c. Appreciation means a full or sympathetic understanding of sb/sth, the feeling of being grateful for sb/sth, or recognizing and enjoying the good qualities of sb/sth. Praise is to use words that express approval or admiration of sb/sth. The difference could be found from the wordings of these two outcomes.

d. We have revised the sentence as suggested.

Line 32: *“The control group was offered the intervention after all the data were collected.”*

e. Primary and secondary outcomes are now separately reported.

**C3.** Introduction: The authors made the introduction more relevant. There is still some restructuring and fine tuning needed.

**R3.** Please find the point-to-point responses below. We have revised the sentences as follows.

a. Line 79: The authors state “despite cultural differences in training and controlling”. However, these differences have not been discussed, it was only stated that training and control is considered positive in China.

a. Line 60: *“Although training and controlling are considered positive in China [1], the high prevalence of child maltreatment is worrying.”*

b. Line 77: Sentence structure is incorrect. Western parents use praise to boost children’s confidence, whereas Chinese parents use praise to ..” or “Western parents tend to use praise in abundance in order to boost their children’s confidence, whereas Chinese parents tend to use praise sparingly to prevent their children becoming arrogant (NB arrogant is just an example).

b. Line 57: *“Western parents tend to use praise in abundance in order to boost their children’s confidence, whereas Chinese parents are apt to apply praise sparingly to prevent their children becoming complacent [3, 4]”*

c. Line 82: Unclear what is meant, please clarify. Is this what the authors meant: “.. more than half of HK parents had used corporal punishment, with 4.5% of these cases resulting in injuries”

c. Line 62: *“A 2006 report on Chinese parents with children aged 18 years or below found more than half of Hong Kong parents had used corporal punishment and nearly 5% of parents had maltreated their children physically, resulting in injuries [6].”*

d. The authors sometimes jump from one topic to the next and the research that is cited does not always apply to the arguments being made. E.g., after talking about physical abuse (until line 89), the authors suddenly start discussing social support.

Line 92: why do the authors talk about students? What is students’ life satisfaction? Children’s satisfaction with their life at school? This can have so many other causes other than parenting. How old were these children?

Again, the use of ‘students being satisfied’ in line 107. Maybe students were not satisfied because of the amount of homework. This needs to be better specified. The following suggestion regarding family support (line 108) implies that the students in line 107 had received family support.

d. We have removed the sentences about social support. We agree with the reviewer that there are other reasons that the students were not satisfied. A more relevant point here is *“In contrast, appreciation, warmth, affection and positive engagement predict children’s social competence and school achievement” (Line 68)*, which we have moved to the end of the first paragraph.

e. Line 110: “yet parents may not know how to praise children appropriately”. Earlier in the introduction the authors talked about praise and parents preferring to be strict. This needs to be combined and some additional literature needs to be reviewed. Perhaps this can be combined with the parenting challenges that Chinese parents experience.

e. We have restructured this paragraph and combined it with the parenting challenges (Introduction 2^nd^ paragraph).

f. Lines 113-117: Good addition of praise types. Please add an example of process praise.

f. We have added an example for process praise.

Line 74: *“Process praise, on the other hand, focuses on the efforts paid or the process (e.g, “Great job! You must have worked very hard.”),…”*

g. Line 127-128: references are needed to back up these statements

g. Reference has been added.

Line 81: *“Parents who held stronger traditional Chinese values were found to have more feelings of shame towards child behaviour problems, and lower intentions to seek help [15].”*

h. Lines 129-133: the authors discuss Triple P as a good example of a universal parenting intervention. This makes the reader wonder why the authors did not use that programme instead, which is well known, effective, and translated into Mandarin. What is the rationale for using the authors’ intervention? With 4 group sessions, Triple P is already pretty brief. Is it too costly (i.e., you do need a qualified triple P facilitator)? Are there too many points of contact with triple P (i.e., 4 group sessions vs only 2 interactive talks)?
For attachment-based interventions research suggests that shorter is better. No such evidence exists for behavioural-based interventions. So why is shorter better? It seems to me that the authors’ intervention is time consuming too, with all the family games to be played over the course of one (or two?) months.

h. Both cost and time are important considerations for public health interventions. We have added more information to explain why we develop a new intervention rather than using Triple P.

Line 93: *“Triple P model was implemented in Hong Kong by the Department of Health as part of its child health service. The programme was delivered by nurses who were supervised by a clinical psychologist, which involves substantial cost to the government and time to the parents [24]. Therefore, shorter community-based public health interventions are worth exploring, but relevant evidence is limited.”*

i. Line 145: to what extent were parents involved in the study design? I see no evidence of CBPR in the current study except for service providers being interviewed. This is not participatory research.

i. The service providers were involved in study design but involvement of the parents was limited to providing feedback on the flow and design of a small trial run of the programme not mentioned in this paper. We figure it may be better for us to remove the phrase to avoid misuse of the term CBPR.

**C4.** Methods
Thanks for providing a rationale for using a clustered design.

**R4.** Thanks for the comments on the Methods. Please find our point-to-point responses below.

a. Line 196: leave out: “The intervention group received the interventions first.” It is sufficient to say that the control group received access to the intervention after data collection was complete.

a. We have removed the sentence “The intervention group received the interventions first.”

b. Line 197: the term follow-up is not appropriate here as there is no actual follow-up assessment (i.e., assessment after the post-intervention assessment). Common description of assessment points are: baseline (Time 1), post-intervention (Time 2), ..-month follow-up (Time 3). Not sure how to describe the authors’ research design, as Time 2 is only after the first part of the intervention.

b. We reorganised the sentence as below.

Line 145: *“We measured all the outcomes at baseline (before the first session of intervention), conducted the second assessment at 1 month (before the second session of intervention) and the third assessment at 3 months.”*

c. Line 203: leave out: “.. no interventions during the assessment period” see my previous point re line 196.

c. We have revised the sentence as suggested.

Line 149: *“The waitlist control group received related services after data collection had been completed.”*

d. Joyful parenting intervention/ SME intervention lines 205-248 - I find the use of both these names confusing
1) What is the name of the actual intervention?
2) Who developed this and how?
3) Based on what?
4) What is the interactive aspect of the talks? They still sound like lectures to me.
5) Is there information available to the public so this study can be replicated?
6) Line 210: praise (positive sharing). The definition of ‘praise’ is not ‘positive sharing’. I think most readers will know what praise is (the expression of approval), so perhaps not necessary to supply a definition here.

7) Regarding ‘Appreciation skills’, I have only ever heard of literary appreciation skills, so not sure what this refers to.
8) Line 236: family games during 4 or 3 weeks? The authors talk about 4 weeks, but then state 21 days. How much paly and practice happened? One or two months?

d. We have revised the section about the Joyful Patenting Intervention as follows.

1) Joyful parenting is the name of the actual intervention. To avoid confusion, we have moved the definition of SME to the Introduction and included more details about the background.

Line 100: *“SME was used as the slogan of the Joyful@HK campaign.”*

Line 106: *“The present trial, Joyful Parenting Pilot Project, was one of the projects in adults under the campaign.”*

2) Line 155: *“The interventions were designed by academic researchers together with community service providers,…”*

3) Line 152: *“The design of the study was based on the concept of SME, Seligman’s PERMA (Positive Emotion, Engagement, Relationships, Meaning and Accomplishment) model [27], and the findings from our previous project about increasing appreciation and reducing criticism in parents [28].”*

4) The games were the interactive parts.

Line 157: *“The contents included skills of giving praise, showing appreciation, and playing enjoyable and interactive family games.”*

Line 164: *“Three simple and interactive family games were led by the social workers, enabling the parents to observe and experience how to give praise or show appreciation.”*

5) The programme rundown has been attached (S3) for replication of the study.

6) The definition of ‘praise (positive sharing)’ has been removed.

7) We rephrased the wordings.

Line 157: *“The contents included skills of giving praise, showing appreciation, and playing enjoyable and interactive family games.”*

8) We apologise for the typo. The practice and family games were documented in one month. It should be 21 times rather than 21 days (in each week).

Line 174: *“Simple worksheets were assigned to the parents to record their practice of three types of praise (specific praise, process praise and outcome praise) and family games they arranged in the following four weeks. Times of praise (each type of praise done in one day was counted as 1 time) and family games in each week were documented.”*

e. Line 217, what does MADD stand for (this has been deleted) – acronyms should be explained at first mentioning.

e. We have added the full name of MADD at first mentioning (moved it to the introduction).

f. Lines 219-220 (reference 29) belongs in the introduction.

f. This has been moved to the introduction.

Line 104: *“The projects in adults aimed to improve the public awareness of Mixed Anxiety and Depressive Disorder (MADD), which was the most common mental disorder in Hong Kong adults (6.9%) [26].”*

g. Lines 226-232: this belongs in the introduction, not in the methods. Please integrate with what is already in the introduction.

g. We would like to keep the detailed description about the intervention in the Methods section. But the definition of process praise was given in the Introduction.

e. The goal of the methods section is to allow for a study to be replicated by someone else. This is currently not possible and more information needs to be provided.
No evidence of any interaction in the ‘interactive talks’. These talks sound like lectures and should not be described as being interactive. The subsequent games do seem interactive as do the practice with focussing on positive traits

e. Please kindly refer to the answers in Methods part d 4)-5). The study can be replicated following the programme rundown (time allocation of each section has been included). We also gave some examples and wordings in the main text. For the interactive process, practitioners could adapt the model to their local communities, such as what interactive games should be selected.

f. There is insufficient evidence of the validity and reliability of the primary outcome measures. How do the authors know their measures assess what they were designed to assess? Any changes found between the control and intervention group could be something else entirely. Retrospective behaviour-related questions (e.g., to quantify the amount of praise) are not necessarily reliable. Plus, as there was no placebo intervention, any changes measured may be due to the effect of attention. These are all aspects that need to be discussed in the limitation section of the study.

The high level of correlation between the different primary outcomes is not necessarily positive as it may be indicative of the different measures assessing the same construct rather than separate constructs. Evidence on some of the secondary outcomes is much stronger as some were based on existing questionnaires.

f. The limitation of the primary outcomes was included in the discussion. However, the outcome measurement could not be changed since the study has been completed. Nevertheless, the qualitative measures provided us extra evidence for the effect of the interventions. We have also added the issue of the comparison group in the limitation.

Line 448: *“First, because validated questionnaires were not available and many existing ones were too long, we developed the outcome and impact-oriented questionnaires to assess the changes in the participants. The measurements showed acceptable to good congeneric reliability. Future studies should test the validity of the praise measurements.”*

Line 455: *“Third, because the target population was healthy and the ‘usual care’ was no care, a waitlist control was adopted. To control for any effect due to attention or interaction, alternative psychological placebos could be used in future studies.”*

g. Measuring children’s perception of the level of parental praise is a great way to make the parent measure of praise stronger. This should be emphasised more, but only if the child questions were very similar to the parent questions.

g. We agree with the reviewer that the children’s feedback is critically important. They may motivate the parents if the programme was proved to be effective in improving children’s performance. But due to the administrative burden, we only included a few questions in the child questionnaire. Since this was a pilot study and our target population was parents, the data in this section may not provide valid findings on the change in children. In the last version of manuscript, we mentioned that *“children aged 12-18 years in the intervention group (n=18) reported significantly higher level of personal health (MD=1.36, 0.14 to 2.58, P=.03), personal happiness (MD=1.33, 0.13 to 2.53, P=.03), and family harmony (MD=1.51, 0.34 to 2.67, P=.01), compared with the children of the same age in the control group (n=29)”*. The intervention might be effective for a specific age range of children. However, this analysis was not prespecified in the protocol and we did not set very strict criteria on the enrollment of the children We have, therefore, removed this part. Hopefully, we can observe more outcomes on children in future similar programmes.

h. Fidelity only checked by the researchers, not by an independent other. How was the quality of the qualitative component ensured?

h. Implementation fidelity is the degree that the programmes were implemented as intended by the programme developers. As our aim was to check whether the programme was delivered following our original rundown, the assessors must be clear about the design. The researchers therefore were eligible to do the fidelity check. The researchers included our project staff, government research officers and senior officers from the collaborator. That was why the evaluation of the first and second talks included 11 observers and 9 observers, respectively. We hope this will ease the concern on the objectivity of the evaluation results.

**C5.** Discussion
Line 503: “.. two simple interactive talks”. What about all the family games that took place over the course of one (or two?) months? It’s not such a brief intervention after all. There are only two points of contact.

**R5.** During the study process, we only sent reminders to the participants to complete the homework (after the first talk). Their own practices were the outcomes rather than the interventions. Therefore, the length of the intervention only included two interactive talks and a release of simple reminders. The worksheets and the provided materials such as the Legos, might be effective components but still were the contents in the interactive talks.
