# Supplementary material for: Promoting positive parenting and mental wellbeing in Hong Kong Chinese parents: A pilot cluster randomised controlled trial
Source: PLoS One. 2022 Jul 20;17(7):e0270064. doi: 10.1371/journal.pone.0270064 (PMC9299310; doi:10.1371/journal.pone.0270064)
Supplement: Attachment — Submitted filename: Response to Reviewers.docx [file pone.0270064.s009.docx]

**Reviewer #2:** The authors did a fantastic job with the revisions and with taking on board all reviewers’ suggestions. The paper has improved immensely over the course of the revision rounds. I just have a few comments (relating to the revised manuscript with the visible track changes). I do not need to review any revisions.

**Authors' Responses to Comments**

**C1.** In the CONSORT diagram it says that all centres and parents allocated to the control group received the intervention. I find this confusing as the control group was a care-as-usual group and only received the intervention after data collection was complete.

**R1.** Thanks very much for your further suggestions. Now we have removed “2 centres (69 parents) received allocated intervention” from figure 1 (CONSORT flow chart).

**C2.** line 93-101:” Parents may benefit from training” would be a more nuanced statement instead of “Parents need training”. Also, if talking about substantial proportions, some kind of evidence on these proportions must be provided. The authors listed some of the stressors parents may have, but not how many parents experience this. Perhaps the easiest way to deal with this is to rephrase the leading sentence in line 93 and not use the words “substantial proportion”.

**R2.** We have revised the sentence as suggested.

Line 77: *“Parents may benefit from training on positive parenting since they experience various child-rearing challenges.”*

**C3.** lines 123-128 – excellent addition to explain the campaign. Perhaps put the words sharing, mind, and enjoyment in between apostrophes, e.g., ‘Sharing’ refers to connecting with family and friends. Because sharing means something else in ‘everyday English’.

**R3.** We have added the apostrophes as suggested.
